# Does Implementation of a Corporate Wellness Initiative Improve Burnout?

**DOI:** 10.5811/westjem.2018.10.39677

**Published:** 2018-11-13

**Authors:** Danielle Hart, Glenn Paetow, Rochelle Zarzar

**Affiliations:** * University of Minnesota Medical School, Hennepin County Medical Center, Department of Emergency Medicine, Minneapolis, Minnesota; † Hennepin County Medical Center, Department of Emergency Medicine, Minneapolis, Minnesota

## Abstract

**Introduction:**

Burnout affects over 50% of all physicians. Nearly 70% of emergency physicians are affected, and it has been found to be as high as 76% in resident physicians overall. Previous wellness initiatives have yielded variable results; therefore, we looked for interventions that could potentially be effective at reversing this trend. We explored effective wellness programs originating from other industries. Our objective was to implement a corporate wellness program with previous evidence of success in other healthcare provider populations. We aimed to investigate whether this program would be effective in decreasing burnout in emergency medicine (EM) residents.

**Methods:**

This program was conducted during required EM resident conference hours from 2016–2017. The Maslach Burnout Inventory was completed before and after the series of sessions, and we collected reactions-level data following completion of the six sessions.

**Results:**

Post-intervention scores revealed a small trend toward increased emotional exhaustion and depersonalization scores, and with increased personal accomplishment scores. The overall satisfaction rating for this program was low, at 1.5 on a 5-point scale. Forty-three percent of residents stated that this intervention subjectively worsened their overall burnout, with another 39% stating it did not improve their burnout at all. A similar trend was seen for effects on wellness.

**Conclusion:**

We found that a corporate wellness intervention that had previously been shown to be successful with other types of healthcare providers did not objectively improve burnout and was subjectively perceived as paradoxically worsening burnout for many residents. This result may be related to the type of intervention chosen (individual vs. systems-focused), the design of the intervention itself, or the unique stressors faced by the resident population. [West J Emerg Med.2019;20(1)138–144.]

## INTRODUCTION

Burnout affects over 50% of all physicians. It is nearing 70% in emergency physicians (EP) and has been found to be as high as 76% in resident physicians. 1–4 Between 2011 and 2014, burnout rates have trended upwards, reaching what has been described as “epidemic proportions.”^1^,^5^ Burnout has been defined as “a syndrome of emotional exhaustion, depersonalization, and a sense of low personal accomplishment that leads to decreased effectiveness at work,” while wellness is an even broader, multidimensional concept. 5,6 Burnout has been shown to have a negative impact on patient safety and quality of care, patient satisfaction, and healthcare costs, as well as having negative effects on the individual such as job dissatisfaction, intent to leave, decreased productivity, and increased incidence of alcohol abuse, depression and suicidal ideation. 7 Various types of wellness, resilience and stress management interventions have been implemented in the past among various populations of healthcare providers, revealing inconsistent results. 8–19

Similar inconsistent results have been seen with corporate wellness programs across a wide variety of industries, with an overall positive impact reported via meta-analysis. 20 While many corporate programs focus on absenteeism and job satisfaction as the main outcomes, one program called “The Happiness Practice” (THP) has shown success for non-physician, emergency medicine (EM)-based healthcare providers (predominantly nurses) in decreasing burnout, increasing wellness, and even increasing patient satisfaction. 20,21 This program has also been shown to decrease burnout in hospital-based executive leadership teams, while increasing individual happiness, resilience, innovation and sustainability [Personal communication, Nancy O’Brien]. THP is focused on methods to increase each individual’s happiness and resilience through helping them develop “new ways of thinking, feeling and behaving that positively impact their life, their work and their environment.” 22

Additional studies are needed to investigate which types of interventions are effective for certain groups of healthcare providers. 15 There is evidence that both systems-based and individual-based wellness initiatives can have benefits. 23 Since previous, individual-focused interventions such as mindfulness and mindful communication have shown improvements in healthcare-provider burnout, we aimed to investigate whether the corporate THP program would be effective to decrease burnout in EM residents. 24–28

## METHODS

We implemented THP for EM residents at an urban training program during regularly scheduled conference hours in 2016–2017. This program included six, monthly, one-hour didactic sessions from September through February, each focusing on a different core principle following an introductory session. These included the following: 1) Be conscious; 2) Honor feelings; 3) Release control in favor for empowerment; 4) Co-create what works now; and 5) Learn life lessons. Sessions were developed and led by two former business executives who co-founded THP. Their company was contracted by our hospital system, and provided this program to any interested groups or units during the period of that contract. Optional, small-group, evening social discussions called “Happy Chats” between sessions were also held at local restaurants between the first three sessions, led by the two co-founders. These included time for building interpersonal relationships and reflecting on the content that was delivered during the conference sessions through facilitated discussion. Following the introductory session, many residents provided feedback that the conference session was too long for the amount of information provided and was not structured in a high-yield format. Therefore, subsequent sessions were adjusted to become 15-minute sessions delivered in a more high-yield fashion. The Maslach Burnout Inventory (MBI) was completed before and after the series of six sessions. Following the completion of all six sessions, residents were also surveyed for reactions-level data using an anonymous electronic platform. 29 Descriptive data are presented, and we conducted thematic analysis using a data-driven inductive approach to code comments. 30 All comments were coded by two independent researchers. The institutional review board determined this study to be exempt.

**Population Health Research Capsule**

What do we already know about this issue?

*Burnout has become prevalent in the medical field, including graduate medical education. Nearly 70% of emergency physicians meet criteria for burnout*.

What was the research question?

Would a corporate wellness program “The Happiness Practice” be effective in decreasing burnout in emergency medicine (EM) residents?

What was the major finding of the study?

*This program was not effective in improving EM resident burnout, despite its prior efficacy with EM nurses and hospital leadership*.

How does this improve population health?

*Specific, individual-focused burnout interventions may not be equally effective among different healthcare provider types. Systems-focused interventions may be beneficial*.

## RESULTS

The response rate for MBI completion was 34 of 46 (74%) residents prior to the training and 24 of 46 (52%) residents after the training. There was a slight trend toward increased overall burnout scores in areas of emotional exhaustion (EE) and depersonalization (DP), with improved mean personal accomplishment (PA) scores following this intervention (Table 1). These trends were also seen in the post-graduate year (PGY)-1 and PGY-2 classes, all with overlapping standard deviations. In the PGY-3+ class, only four respondents completed the post-intervention MBI; thus, these results should be interpreted with caution (Table 1, Figure). The response rate for the reactions-level data post-survey was 23 of 46 residents (50%). Results of the survey questions are in Table 2. The overall satisfaction rating was 1.5 on a 5-point scale (Table 2). An average of 27 residents attended each conference session.

Nineteen individuals provided free-text comments on the survey. There were only two positively coded responses and 17 negatively coded responses, with 100% rater agreement on these categorizations. One positively coded comment revolved around sound philosophy and the other reflected enthusiastic delivery. Thematic analysis of negatively coded comments initially had 84% agreement between raters, with 100% agreement after discussion. Some responses included multiple comments touching on various themes. The following themes emerged: a) The instructors had a poor understanding of residency stressors, resulting in lack of relevance (3); b) the instructors had a poor understanding of EM work, resulting in a lack of relevance (4); c) the sessions needed to be tailored better to healthcare professionals overall (4); d) the sessions were generally unhelpful (11); and e) residents would prefer to be learning topics related to medicine during conference hours (3).

One resident commented, “*A medicine topic lecture would have been more helpful because part of my sensation of burnout is that I do not have enough time to study and learn things, and the anxiety that produces leads to more burnout*.” Another commented, “*The situations they brought up as things that were stressful (e.g., mild workplace disagreements) didn’t seem relevant to emergency physicians/residents. I remember sitting in the session and thinking, “I’m not stressed because some co-worker said something mildly bothersome. I’m stressed because I’m overworked and trying to prevent people from dying every day at work*.”

## DISCUSSION

This corporate-based wellness intervention, which has been successful for other types of healthcare providers, did not appear to have much of an effect on overall burnout levels on the MBI and was quite negatively perceived by EM residents. 21 PGY-1s and PGY-2s had a trend towards worsening EE and DP levels during the course of the study. Previous studies have found that burnout and depression, including EE and DP scores, increase significantly throughout intern year, and empathetic concern decreases, likely due to challenging clinical experiences and workloads, long work hours and increased sleep deprivation, limited time to nurture personal lives, and other factors. 31–35 Although an increase in burnout has been found from the start of intern year to mid-year or end of year, further worsening of burnout has not been found between mid-year and end of year in interns or in subsequent years of training. 31,32,34,35 Our post-EE, DP and PA scores, collected nine months into intern year, were similar to end-of-year intern EE and PA scores in pediatric residents and DP scores in internal medicine residents from prior studies.^32^,^35^

It is unknown whether our intervention had any effect on this trend towards worsening EE and DP, or was just not powerful enough to reverse it. It is possible that any positive impacts of this intervention on burnout could be masked by the expected increase in burnout during intern year, with sustained burnout in subsequent years of training. However, the fact that 82% of residents subjectively felt that the intervention either did not improve or worsened their burnout makes a masked positive effect seem less likely, as does the trend towards worsening burnout scores in our PGY-2 cohort.

The PGY-1 class did experience an improvement in their PA. It is again unknown how much of this stemmed from this intervention vs. other factors such as the increase in confidence and competence that often occurs during PGY-1 year. Since the PGY-3+ class only had four individuals (<25%) complete the post-intervention MBI, the results for this subgroup must be interpreted with caution, as a large potential for bias exists. While some benefit has been seen with individual-focused initiatives to improve burnout (such as mindfulness and small-group activities focusing on meaning and interconnectedness), recent literature has begun to focus more on the importance of systems-based initiatives to decrease burnout and improve wellness. 7,15,23,36–40 It seems that interventions in both of these areas may be helpful to move the needle in a meaningful way on the burnout and wellness spectrum.

When considering why this intervention was successful in other groups of healthcare workers (ED nurses and hospital leadership) but not EM residents, it is possible that individuals in residency training represent a unique population because 1) residents have longer work hours than other healthcare provider groups, often working 60–80 hours per week; 2) a contributing factor to lack of resident and physician wellness is not having enough time away from work to nurture aspects of one’s personal life and spend time with family and friends, and therefore they want to perceive time spent at work as high yield and well spent; 11,41,42 3) residents are learners, trying to develop competence in a medical specialty and so have an increased focus on developing individual competence with less of a focus in other areas; 4) residents could be living in more of a moment-to-moment mindset and may not see the long-term benefit of such sessions; and 5) burnout has been shown to increase over the first year of residency.^31^–^35^, ^42^ Their perceptions of this wellness intervention worsening burnout could be related to these factors.

Additionally, THP has a proprietary “Return on Happiness” system that is used to track and report participant progress. That system reportedly “measures and reports qualitative and quantitative improvements at individual, group and organizational levels” (http://www.experiencehappiness.biz/thp/). In this study, we elected not to use this proprietary evaluation tool and instead used the MBI in conjunction with a newly developed reactions-level survey. Our use of a different evaluation tool may also have contributed to the discrepancy between the previously reported success of this intervention and our results.

Our results suggest that wellness initiatives for residents may be perceived as more beneficial if they were more focused on specific EP-related stressors and coping techniques and taught by individuals such as EPs (with expertise in burnout and wellness) who understand EM challenges well. Since residents did not seem to perceive this information to be important, a more explicit explanation of how wellness relates to the residents’ general underlying goal of becoming a competent and satisfied physician, and how burnout impacts patient quality of care and safety, patient satisfaction and healthcare costs, may be required. 7

Positioning this wellness intervention during mandatory educational time was also negatively received. While we feel that education on “non-medicine” topics such as communication, professionalism, wellness/burnout/depression, and other topics are important, it is possible that certain types of wellness activities may work better in optional settings, allowing residents autonomy to choose whether they would like to participate. 11,43 Optional sessions could, however, result in individuals with high levels of burnout or low levels of wellness who could benefit from the sessions choosing not to attend.

Since EM has the highest rate of burnout of any medical specialty, nearing 70%, we feel that efforts to elucidate effective interventions for EM providers and residents are important. 3 Conducting a needs assessment within individual residency programs to elicit what that program’s residents feel would truly help their wellness would likely also be of benefit, allowing this information to guide the content of future interventions.^23^ Exploring system-based frustrations and inefficiencies and having the EM residents partner with departmental leadership to identify systems issues that could be improved may also be of benefit. 37,38

## LIMITATIONS

This study examined THP at only a single residency program over a one-year period. The response rates for the post-intervention MBI and survey were 50%–52%, which could be a source of bias. The lower number of PGY-3+ residents completing the post-intervention measures could also significantly bias these subgroup results. Even though conference time is “protected,” due to logistics with resident schedules and off-service rotations not all residents were able to attend each session. It is possible that the results would have been different if every person could attend each session. These sessions were also integrated into our required, resident conference hours; it is unclear if prior successes of the THP program included voluntary or mandatory participation.

Based on resident feedback, the length of each session was decreased from 60 minutes to 15 minutes; therefore, the implemented program was different than that initially described by O’Brien et al.^21^ This could have affected the effectiveness of this intervention. Additional demographics were not collected outside of PGY, not allowing further investigation using these variables. Finally, there was no control group for this study, and as far as we are aware, there is no literature describing expected trends in burnout throughout EM residency specifically. Therefore, while THP was not powerful enough to reverse this natural trend towards increased burnout seen in intern year, it is impossible to know if it had any smaller positive or negative effects on the rate of burnout progression.

## CONCLUSION

We found that a corporate wellness intervention that had previously been shown to be successful with other types of healthcare providers did not objectively improve burnout and was subjectively perceived as paradoxically worsening burnout for many residents. This may be related to the type of intervention chosen (individual vs. systems focused), the design of the intervention itself, or unique stressors faced by the resident population.

## Figures and Tables

**Figure:**
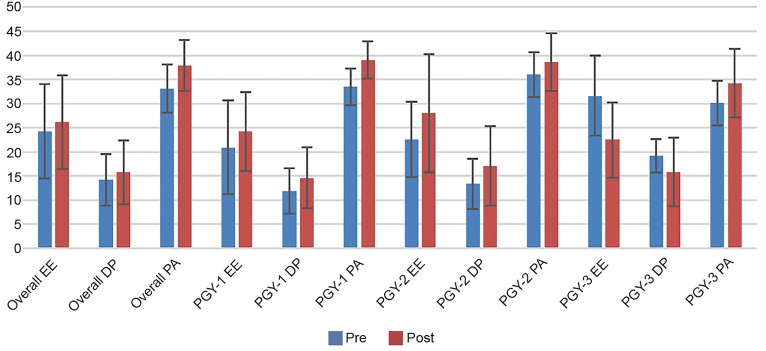
Maslach Burnout Inventory scores of EM residents before and after “The Happiness Practice.” PGY-3+ also includes EM/IM combined residents in their third fourth and fifth years. Pre = prior to implementation of “The Happiness Practice” sessions, Post = after completion of these six sessions. The PGY-3 class only had four respondents post-intervention; so, these results should be interpreted with caution. *PGY*, post-graduate year; *EM*, emergency medicine; *MBI*, Maslach Burnout Inventory; *EE*, emotional exhaustion; *DP*, depersonalization; *PA*, personal accomplishment.

**Table 1:** Maslach Burnout Inventory scores of emergency medicine residents before and after participation in a corporate wellness program.

	Pre-EE mean (SD)	Post-EE mean (SD)	Pre-DP mean (SD)	Post-DP mean (SD)	Pre-PA mean (SD)	Post-PA mean (SD)
All classes combined (n=34 pre-intervention, n=24 post-intervention)						
MBI score	24.3 (9.8)	26.2 (9.7)	14.2 (5.4)	15.8 (6.6)	33.1 (5.0)	37.9 (5.3)
PGY-1 class (n=13 pre-intervention, n=9 post-intervention)						
MBI score	20.9 (9.7)	24.2 (8.2)	11.9 (4.7)	14.6 (6.3)	33.5 (3.8)	39.0 (3.9)
PGY-2 class (n=9 pre-intervention, n=7 post-intervention)						
MBI score	22.6 (7.8)	28 (12.2)	13.4 (5.2)	17.1 (8.2)	36.0 (4.7)	38.6 (6.0)
PGY-3+ class (n=9 pre-intervention, n=4 post-intervention)						
MBI score	31.6 (8.3)	22.5 (7.8)	19.2 (3.4)	15.8 (7.1)	30.1 (4.7)	34.25 (7.1)

PGY-3+ also includes EM/IM combined residents in their third, fourth, and fifth years. There are 46 total residents in this training program, 14 per year, and two per class in the fourth and fifth years of EM/IM.

Pre = prior to implementation of the happiness practice sessions, Post = after completion of these 6 sessions.

Three learners did not identify their learner level on the pre-intervention MBI, and four did not identify their learner level on the post-intervention MBI.

Scoring for EE (per MBI scoring guidelines): High = 27 or greater, Moderate = 17–27, Low = 0–16.

Scoring for DP (per MBI scoring guidelines): High = 13 or greater, Moderate = 7–12, Low = 0–6.

Scoring for PA (per MBI scoring guidelines): High burnout = 0–31, Moderate burnout = 32–38, Low burnout = 39 or greater. Lower personal accomplishment raw scores are an indication of higher burnout, so this item is reverse scored.

*PGY*, post graduate year; *EM*, emergency medicine; *IM*, internal medicine; *MBI*, Maslach Burnout Inventory; *EE*, emotional exhaustion; *DP*, depersonalization; *PA*, personal accomplishment.

**Table 2:** Survey questions and residents’ responses.

Likert scale rating	% of residents
Question #1: Please rate your overall satisfaction with “The Happiness Practice.”	
1 = low satisfaction	70%
2	13%
3 = average satisfaction	17%
4	0%
5 = high satisfaction	0%
Question #2: How much do you feel this series improved (lessened) your overall burnout?	
0 = “it made my burnout worse”	43%
1 = not at all	39%
2	9%
3 = moderately	9%
4	0%
5 = significantly	0%
Question #3: How much do you feel this series improved your overall wellness?	
0 = “it made my wellness worse”	17%
1 = not at all	57%
2	17%
3 = moderately	9%
4	0%
5 = significantly	0%

Resident survey responses after implementation of “The Happiness Practice.”
